# SPTBN2 regulated by miR-424-5p promotes endometrial cancer progression via CLDN4/PI3K/AKT axis

**DOI:** 10.1038/s41420-021-00776-7

**Published:** 2021-12-09

**Authors:** Pengling Wang, Ting Liu, Zhendan Zhao, Zhiling Wang, Shujie Liu, Xingsheng Yang

**Affiliations:** Department of Obstetrics and Gynecology, Qilu Hospital of Shandong University, Jinan, Shandong 250012 People’s Republic of China

**Keywords:** Endometrial cancer, Endometrial cancer

## Abstract

Endometrioid Endometrial Cancer (EEC) is the main subtype of endometrial cancer. In our study, we demonstrated that SPTBN2 was significantly overexpressed in EEC tissues. Upregulated SPTBN2 expression was positively associated with poor prognosis. In addition, we testified that SPTBN2 knockdown significantly inhibited the proliferation, migration, and invasion of EEC cells. Moreover, we found SPTBN2 could interact with CLDN4 to promote endometrial cancer metastasis via PI3K/AKT pathway. Then we further demonstrated that CLDN4 is upregulated in EEC and promotes EEC metastasis. CLDN4 overexpression could partially reversed the decrease in cell migration and invasion caused by SPTBN2 downregulation. In addition, we confirmed that SPTBN2 was a target of miR-424-5p, which plays a tumor suppressor in endometrial cancer. Rescue experiments showed that inhibition of SPTBN2 could partially reverse the effect of miR-424-5p in EEC. In conclusion, we demonstrated that by acting as a significant target of miR-424-5p, SPTBN2 could interact with CLDN4 to promote endometrial cancer metastasis via PI3K/AKT pathway in EEC. Our study revealed the prognostic and metastatic effects of SPTBN2 in EEC, suggesting that SPTBN2 could serve as a prognostic biomarker and a target for metastasis therapy.

## Introduction

Uterine corpus endometrial carcinoma (UCEC) is a group of epithelial malignant tumors occurring in the endometrium. It is the most common gynecological tumor in developed countries, and its prevalence is on the rise [1]. It is estimated that there were 61,880 new cases and 12,160 deaths people in the United States in 2019 [2]. By 2020, More than 410,000 cases were diagnosed worldwide, accounting for 4.5% of all new cases [3], and ~65,620 people were diagnosed in the United States with ~12,590 deaths [4]. Endometrial cancer is the fourth most common cancer in women (7% of cancers in women), and the sixth most common cause of cancer death in the United States [4].

Bokhman proposed the classification of endometrial cancer subtypes in 1983 [5]. UCEC can be divided into two general subtypes: type I (endometrioid carcinoma) and type II (non-endometrioid carcinoma). Type I is estrogen-dependent and has the characteristics of estrogen-related, better differentiation, and good prognosis. Type II is estrogen-independent, has no clear relationship with estrogen, poor differentiation, and poor prognosis. A recent study suggests that the rise in endometrial cancer is driven by non-endometrioid subtypes, which are not as strongly associated with obesity as endometrioid cancer [6]. However, regardless of these two types, early detection and treatment could lead to a better long-term prognosis. Therefore, identifying the molecular mechanisms of UCEC genesis and development will facilitate diagnosis and treatment to improve UCEC overall survival.

SPTBN2, a major component of the cytoskeleton of cell membranes and consists of two α and two β spectrin subunits. As a protein-coding gene, the protein products of SPTBN2 belong to the Spectrin family. Studies have shown that SPTBN2 is up-regulated in MPNST [7], colorectal cancer [8], and ovarian cancer [9],which was involved in a variety of biological processes related to tumorigenesis. SPTBN2 has been identified as one of the marker genes for the pathogenesis of cancer and plays an important role in the pathogenesis of cancer, which is of great significance for understanding the pathogenesis and early diagnosis of cancer [10]. However, the expression, function, and molecular mechanism of SPTBN2 in endometrial cancer remain unclear.

In epithelial tissues, intercellular interactions are mediated by junctional complexes consisting of tight junctions (TJ), adherent junctions (AJ), desmosomes, and gap junctions [11]. AJs are responsible for mechanical adhesion between adjacent cells, while TJs play a key role in maintaining tissue homeostasis and maintaining cell polarity [12]. The loss of these connective complexes may result in the malignant transformation of epithelial cells. Loss of TJ leads to loss of cell polarity and increased motility and aggressiveness of cells [13]. Claudins are composed of a multigene family of membrane proteins that play an important role in the formation and maintenance of TJ [14]. Studies have shown that claudin-4 (CLDN4) is highly expressed in ovarian cancer [15], gastric cancer [16], breast cancer [17], and hepatocellular carcinoma [18]. What’s more, CLDN4 has been shown to enhance cell invasion and metastasis by promoting EMT [18–20]. Wang et al. have shown that CLDN4 is aberrantly expressed in breast cancer cells and contributes to cell migration and invasion [17]. Landers et al. found CLDN4 was upregulated in both primary and metastatic tumor specimens compared with benign prostatic hyperplasia at both RNA and protein levels, and CLDN4 may be useful as a potential marker and therapeutic target for prostate metastases [21]. Luo et al. confirmed the expression of CLDN4 in gastric cancer tissues and cell lines was significantly lower than that in adjacent normal tissues or gastric epithelial cells. Moreover, CLDN4 may inhibit the proliferation, migration, invasion, and tumogenesis of gastric cancer cells by inactivating the PI3K/Akt signaling pathway, and enhancing the sensitivity of gastric cancer cells to chemotherapy [22]. However, the molecular mechanism of CLDN4 in EEC remains unclear.

In this study, we investigated the expression and the clinical values of SPTBN2 in endometrial cancer, and we mainly explored the biological functions and the potential interaction among miR-424-5p, SPTBN2 and CLDN4, and identified their effects on progression in endometrial cancer. Our results indicated that SPTBN2 served as an oncogene in endometrial cancer. The potential mechanism could partly explain the reason for the highly expressed SPTBN2 in the promotion of endometrial cancer occurrence and development. Our study could provide a theoretical basis for the precise treatment of UCEC patients.

## Materials and methods

### Patients and tissue specimens

A total of 24 paired fresh EEC tissues and normal tissues were collected from EEC patients who underwent primary surgery in Qilu Hospital of Shandong University from 2017 to 2020. A total of 60 paraffin-embedded EEC samples and 25 endometrial normal samples were collected from the Pathology Department of Qilu Hospital of Shandong University between 2012 and 2018. All patients were diagnosed with EEC by pathology. All experiments in this study were approved and supervised by the Ethics Committee of the Qilu Hospital of Shandong University. And the informed consent was signed by all patients.

### Statistical analysis

All experiments were repeated three times independently, and data are shown as the means ± SEMs. Statistical comparison between two and more than two groups was performed using Student’s *t*-test or One-way ANOVA, respectively by SPSS v22.0 (USA) and GraphPad Prism 8.01 (USA). The survival analysis was conducted by the Kaplan–Meier method with a log-rank test. Two-tailed Spearman correlation analysis was used to analyze the correlation between SPTBN2 expression with clinicopathological features and possible upstream or downstream molecules. Images were processed using GraphPad Prism 8.01 and Photoshop CS6 (USA). Differences were considered statistically significant when *p* < 0.05 (*p < 0.05, **p < 0.01, ***p < 0.001, ****p < 0.0001).

### Supplemental methods

The reagents and protocols about qRT-PCR, IHC, Western blot, flow cytometry, Immunoprecipitation, Luciferase reporter assay, and in vitro and in vivo functional assays of UCEC cells were described in detail in the supplementary materials and methods. Primer sequences are listed in supplementary Table 1.

## Results

### SPTBN2 expression was frequently upregulated and correlated to overall survival time in endometrial cancer

**Fig. 1:**
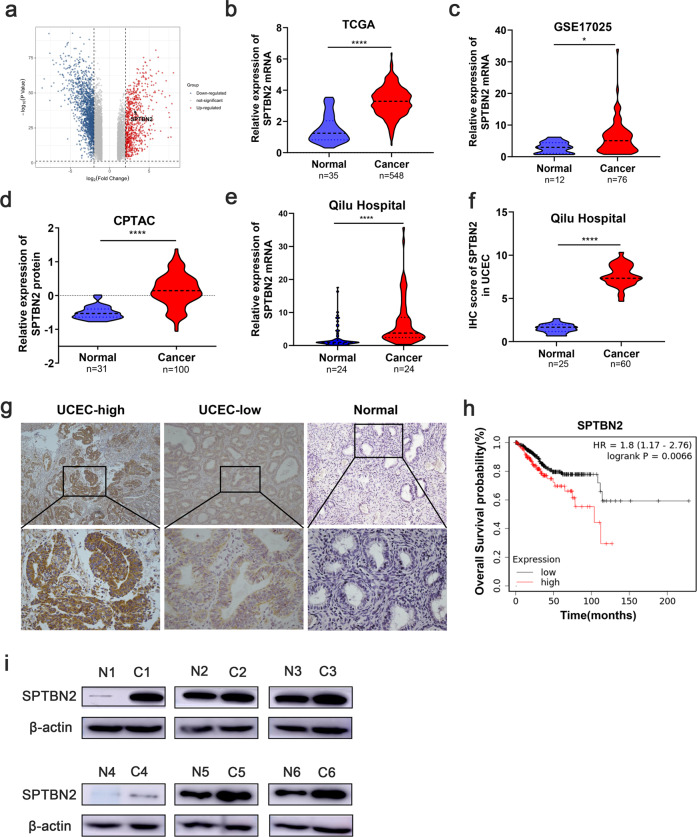
Expression and prognostic significance of SPTBN2 in UCEC. **a** Volcano of differential genes in UCEC and nontumor tissues from TCGA UCEC database. **b**, **c** SPTBN2 mRNA expression analysis in UCEC and nontumor tissues in TCGA database (**b**) and GSE17025 (**c**). **d** SPTBN2 protein expression analysis according to CPTAC database. **e** The mRNA expression of SPTBN2 in 24 paired EEC tissues and adjacent normal tissues. **f**, **g** Representative IHC images of SPTBN2 expression in EEC and normal tissue (**g**). IHC score for SPTBN2 in 60 EEC samples and 25 endometrial normal samples (**f**). **h** High expression of SPTBN2 was associated with poor overall survival of UCEC patients based on TCGA database. **i** SPTBN2 protein expression was detected by western blot in six paired EEC tissues and adjacent normal tissues from EEC patients. C1-C6, represented EEC tissues; N1-N6, represented adjacent normal tissues. (*p < 0.05, **p < 0.01, ***p < 0.001, ****P < 0.0001. The data expressed as the mean ± SD).

Differential gene expression was analyzed in TCGA UCEC database, SPTBN2 was found significantly overexpressed in UCEC, log2(FC) = 3.176, *p* = 1.4E-33 (Fig. 1a). Then we carried out an analysis of TCGA UCEC database (Fig. 1b) and GSE17025 (Fig. 1c), found the mRNA expression of SPTBN2 was upregulated in UCEC tissues compared to normal tissues. Consistently, the protein expression of SPTBN2 was elevated in UCEC tissues in the CPTAC database (Fig. 1d). To investigate the potential role of SPTBN2 in human UCEC pathogenesis, we further verified SPTBN2 expression in EEC tissues using qRT-PCR, western blot, and IHC. Results showed that the mRNA level of SPTBN2 in 24 paired fresh EEC tissues was frequently upregulated, compared to the normal tissues (Fig. 1e). The same as the protein level (Fig. 1f, g, i). Meanwhile, Kaplan–Meier survival analysis indicated that UCEC patients with higher SPTBN2 expression(*n* = 137) appeared to have a shorter Overall Survival(OS) probability compared with those patients who expressed lower levels of SPTBN2(*n* = 406) (*p* < 0.01, Fig. 1h). Then we analyzed the relationship between clinicopathological parameters and SPTBN2 expression in EEC patients. Results showed that the higher expression of SPTBN2 had significantly related to prognosis, but no age, FIGO stage, Differentiation grade, and lymph node status (supplementary Table 2), which may be affected by sample size.

### SPTBN2 promotes endometrial cancer cell proliferation, migration, and invasion in vitro and in vivo

**Fig. 2:**
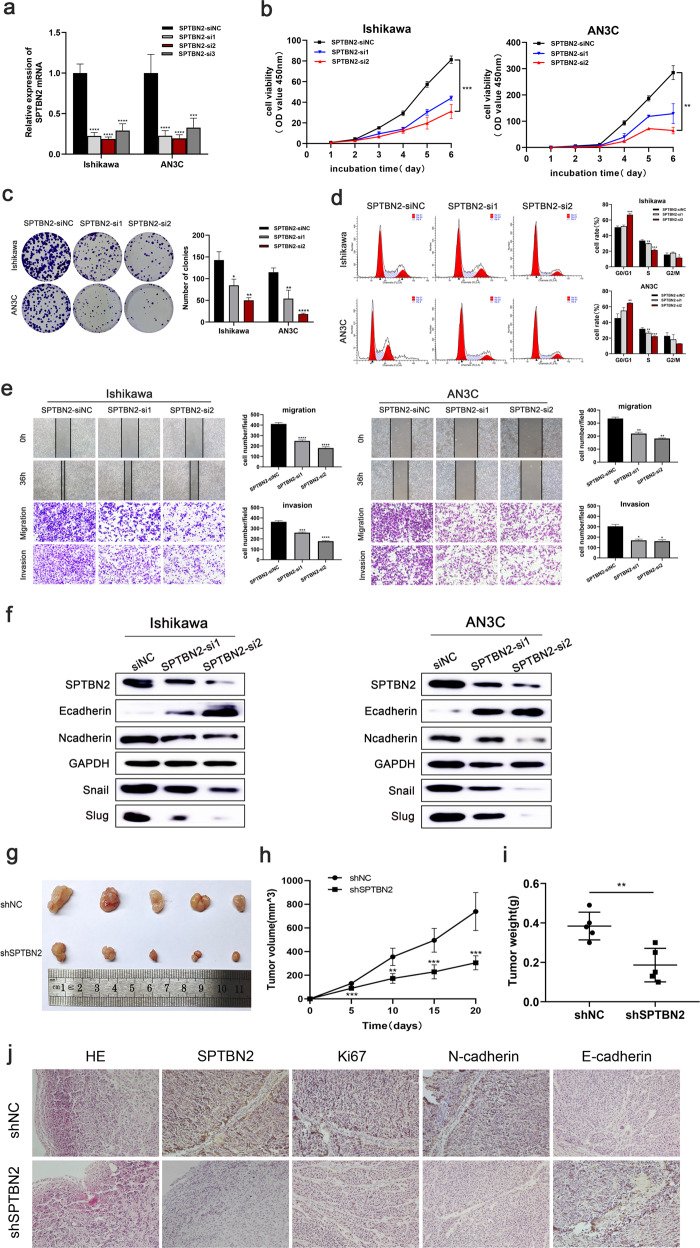
SPTBN2 promotes UCEC cell proliferation, migration, and invasion in vitro and in vivo. **a** The transfection efficiency was determined by qRT-PCR. **b, c** CCK8 and Colony formation assays were performed to identify proliferation after SPTBN2 knockdown in UCEC cells. **d** Downregulation of SPTBN2 promoted G0/G1 cell cycle arrest was detected by flow cytometry. **e** Wound healing and Transwell assays were performed to identify metastasis ability after SPTBN2 knockdown in UCEC cells. **f** The changes of EMT biomarkers expression after SPTBN2 knockdown were detected by Western blot. **g** Images of the subcutaneous xenografts from different groups of nude mice transfected with sh-NC, sh-SPTBN2 respectively. **h** Tumor volumes was calculated after injection every 5 days and tumor growth curves were conducted. **i** Tumor weights were lower for xenograft tumors with SPTBN2 knockdown than for xenograft tumors with shNC. **j** Representative images of IHC staining of Ki67, N-cadherin, and E-cadherin in xenograft tumors. (*p < 0.05,**p < 0.01, ***p < 0.001,****p < 0.0001. The data expressed as the mean ± SD).

Since the effects of SPTBN2 on UCEC had never been reported before, we investigated the biological function of SPTBN2 in UCEC next. We transfected three sequences of SPTBN2 targeting siRNA into Ishikawa and AN3C cells and detected the transfection efficiency by qRT-PCR (Fig. 2a). To further determine whether SPTBN2 is related with proliferation, migration, and invasion in UCEC cells, a series of molecular functional experiments was conducted. The growth curves detected by the CCK8 assay showed knockdown of SPTBN2 could significantly inhibit the growth in UCEC cells (Fig. 2b). And the result of the colony formation assay presented that the silence of SPTBN2 resulted in reduced colonies in UCEC cells (Fig. 2c) which was consistent with the results of CCK8 assay. These findings indicated that SPTBN2 promoted cell growth in UCEC. As we all know, cell cycle arrest is an important factor in inhibiting tumor cell proliferation. Therefore, we evaluated the effect of silencing SPTBN2 on the characteristic by flow cytometry analysis in UCEC cells. Cell cycle assay showed that knockdown of SPTBN2 in UCEC cells resulted in a gradual increase in the number of cells in G0/G1 phase and a gradual decrease in the number of cells in the S phase (Fig. 2d), which was confirmed that cell cycle arrest occurred in G1 phase after silencing SPTBN2. Otherwise, Wound healing and Transwell assays were performed to show that SPTBN2 depletion inhibited the ability of cell migration and invasion in UCEC(Fig. 2e). Besides, Western blot showed that the expression of N-cadherin, Snail, and Slug were downregulated, while E-cadherin was upregulated by SPTBN2 knockdown in UCEC cells (Fig. 2f). All in all, these data indicated SPTBN2 might be involved in tumor metastasis.

In vivo experiment was performed to illustrate the oncogenic role of SPTBN2. Firstly, we established SPTBN2-knockdown and empty vector-transfected Ishikawa cell line, then subcutaneously injected the cells into the armpit of 4 weeks female nude mices; therefore, an engrafted tumor mouse model was established. It was obvious that the tumor volume in the SPTBN2-knockdown group was significantly reduced compared to the control groups (Fig. 2g, h), and the tumor weight in the SPTBN2 silencing group was also significantly reduced (Fig. 2i). Meanwhile, we performed IHC staining proved that the expression of Ki67 and N-cadherin in the SPTBN2-knockdown group was significantly lower than that in the control group, while the expression of E-cadherin in the SPTBN2-knockdown group was significantly elevated (Fig. 2j). Overall, there is no doubt that these findings illustrated the oncogenic role of SPTBN2 in endometrial cancer.

### CLDN4 was a downstream target of SPTBN2 in endometrial cancer

**Fig. 3:**
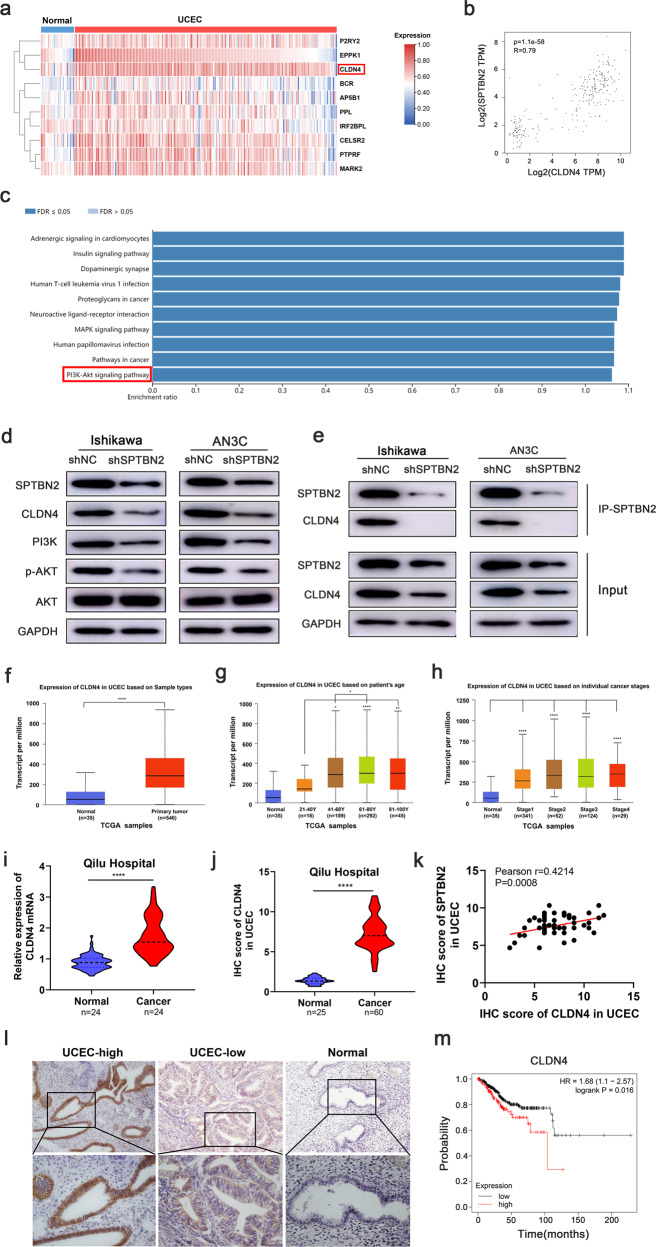
CLDN4 is a downstream target of SPTBN2 in UCEC. **a** The top ten genes with the best positive correlation were revealed through co-expression analysis with SPTBN2 in the TCGA UCEC database. **b** A positive correlation between SPTBN2 and CLDN4 was found in the TCGA database and starBase-database. **c** KEGG analysis indicated that CLDN4 expression was closely correlated with the expression of PI3K/AKT pathway gene sets by WEB-based Gene Set Analysis Toolkit (http://www.webgestalt.org/). **d** The protein expression of CLDN4 and the genes related to PI3K/AKT pathway were examined in UCEC cells after SPTBN2 knockdown. **e** Proteins extracted from UCEC cells with SPTBN2 knockdown were incubated with antibody for SPTBN2 and subjected to IP/Western blot to investigate the interaction. **f** The mRNA expression of CLDN4 in UCEC and normal tissues from TCGA datasets. **g**, **h** Expression of CLDN4 in UCEC based on patient’s age and individual cancer stages from TCGA datasets. **i** The mRNA expression of CLDN4 in 24 paired UCEC tissues and normal tissues. **j**, **l** Representative IHC images of CLDN4 expression in UCEC and normal tissues(**l**), IHC score for CLDN4 in the clinical tissues (**j**). **k** Correlation between SPTBN2 and CLDN4 in UCEC tissues. **m** Kaplan–Meier analysis of UCEC cohorts based on predictive survival analysis. (*p < 0.05, **p < 0.01, ***p < 0.001, ****p < 0.0001. The data expressed as the mean ± SD).

To further investigate the molecular mechanisms of SPTBN2 induced tumorigenesis in endometrial cancer, we conducted co-expression analysis with SPTBN2 and obtained the top ten genes with the best positive correlation according to the mRNA expression of SPTBN2 in TCGA datasets (Fig. 3a). Moreover, we found SPTBN2 expression was positively correlated with CLDN4 expression in UCEC from GEPIA analysis (http://gepia.cancer-pku.cn/detail.php) (Fig. 3b). UCEC patients with high SPTBN2 expression were frequently accompanied with the high expression of CLDN4. WEB-based Gene Set Analysis Toolkit (http://www.webgestalt.org/) analysis indicated that pathway involved in PI3K/AKT was activated in cases with high CLDN4 expression (Fig. 3c). Next, the western blot indicated that knock-down SPTBN2 decreased the expression of CLDN4 and key molecules in PI3K/AKT pathway in UCEC cells (Fig. 3d). Blocking SPTBN2 during the culture of UCEC cells reduced CLDN4 expression. Immunoprecipitation (IP) assay further indicated that SPTBN2 could interact with CLDN4 to form an SPTBN2/CLDN4 complex. After SPTBN2 knockdown, the SPTBN2/CLDN4 complex was reduced. (Fig. 3e).

Next, we investigated CLDN4 expression in endometrial cancer and normal tissues using transcriptome data downloaded from TCGA datasets and discovered that CLDN4 exhibited higher expression in endometrial cancer than in normal tissues (Fig. 3f). What’s more, CLDN4 expression was associated with the patient’s age and cancer stage based on TCGA datasets (Fig. 3g, h). Then we verified the expression of CLDN4 in 24 paired EEC tissues which was upregulated frequently, compared to the normal tissues, using qRT-PCR (Fig. 3i). Consistently, results of IHC staining indicated that CLDN4 in 60 EEC patients had higher expression than in normal tissues (Fig. 3j, l) and was positively related with SPTBN2 in EEC (*r* = 0.42, Fig. 3k). What’s more, Kaplan–Meier survival analysis indicated that endometrial cancer patients with higher CLDN4 expression appeared to have a shorter survival probability compared with those patients who expressed lower levels of CLDN4 (Fig. 3m). The relationship between clinicopathological parameters and CLDN4 expression in EEC patients suggests that higher expression of CLDN4 was associated with prognosis (supplementary Table 3).

**Fig. 4:**
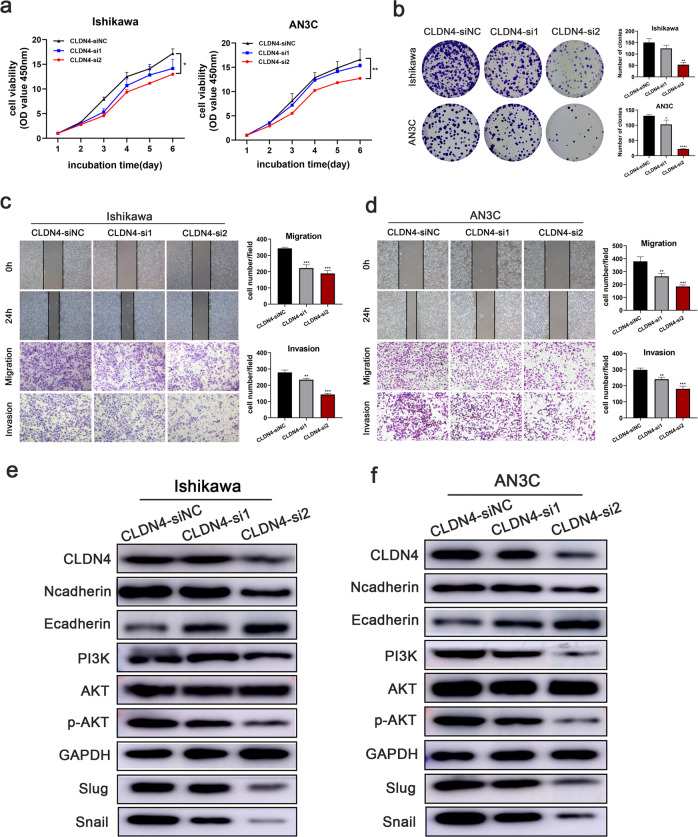
CLDN4 promotes UCEC cell proliferation, migration, and invasion in vitro. **a**, **b** CCK8 and EDU assays were performed to identify proliferation after CLDN4 knockdown in UCEC cells. **c**, **d** Wound healing and Transwell assays were performed to identify metastasis ability after CLDN4 knockdown. **e**, **f** Changes in the expression of EMT biomarkers and the genes related to PI3K/AKT pathway after CLDN4 knockdown in UCEC cells were detected by Western blot. (*p < 0.05, **p < 0.01, ***p < 0.001, ****p < 0.0001. The data expressed as the mean ± SD).

Because the function of CLDN4 was unclear in endometrial cancer, cells were transfected with two sequences of siRNAs against CLDN4. Then we found that CLDN4 knockdown altered the growth (Fig. 4a, b) and metastasis (Fig. 4c, d) of UCEC cells. Moreover, the expression of N-cadherin, Snail, and Slug were downregulated while E-cadherin was upregulated, and PI3K/AKT pathway was blocked in experimental cells verified by western blot (Fig. 4e, f).

### SPTBN2 activated the PI3K/AKT signaling pathway through CLDN4

**Fig. 5:**
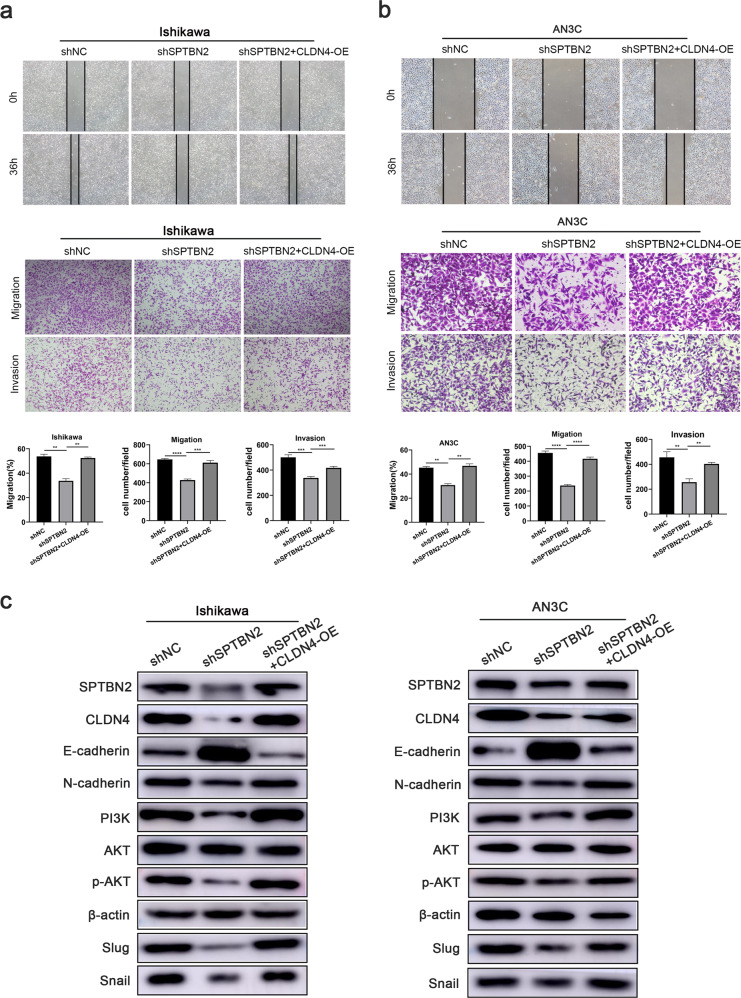
SPTBN2 activates the PI3K/AKT signaling pathways through CLDN4. **a**, **b** Wound healing and Transwell assays of the rescue experiment in UCEC cells. **c** Western blot analysis of PI3K/AKT signaling pathway and EMT-related markers of the rescue experiment in UCEC cells. (*p < 0.05, **p < 0.01, ***p < 0.001, ****p < 0.0001. The data expressed as the mean ± SD).

Rescue experiments were performed to verify whether CLDN4 was involved in SPTBN2 mediated metastasis effects on UCEC cells. We knockdown SPTBN2 and overexpressed CLDN4 in UCEC cells simultaneously and discovered that overexpression of CLDN4 rescinded the reduced cell migration and invasion induced by SPTBN2 knockdown (Fig. 5a–c). These results suggested that CLDN4 was an important gene to promote UCEC metastasis.

### SPTBN2 is negatively regulated by miR-424-5p in endometrial cancer

**Fig. 6:**
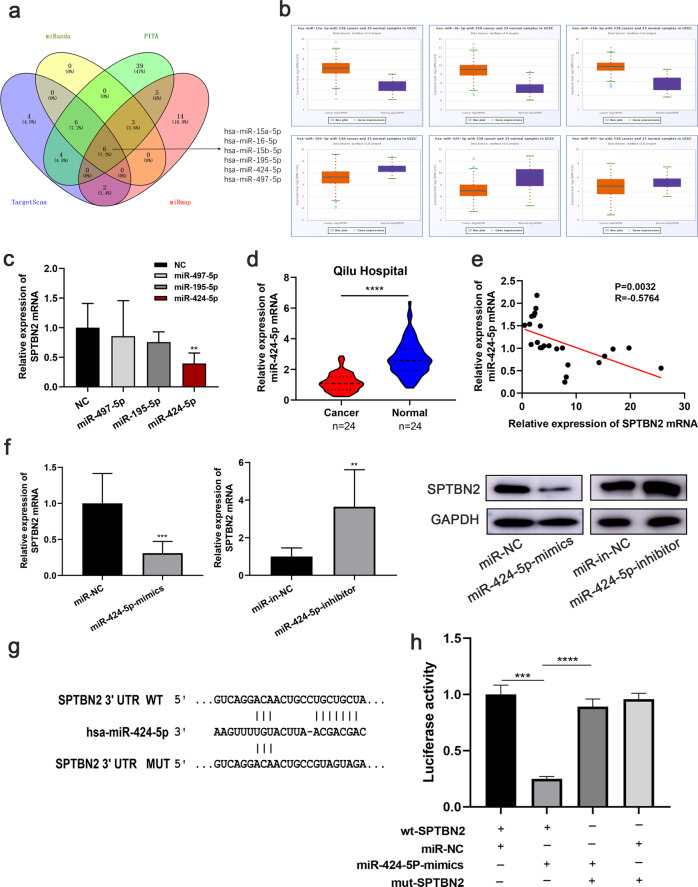
SPTBN2 is a direct target of miR-424-5P. **a**, **b** Screening database speculated that the 3'-UTR binding site of SPTBN2 might bind to the related miRNA. **c** SPTBN2 expression after transfected with miR-195-5p mimics, miR-497-5p mimics, and miR-424-5p mimics in Ishikawa cell were detected by qRT-PCR. **d** The expression of miR-424-5p in 24 paired UCEC tissues and normal tissues. **e** The correlation between SPTBN2 and miR-424-5p in 24 UCEC tissues. **f** The mRNA and protein expression of SPTBN2 in Ishikawa cells was determined by qRT-PCR and Western blot after transfected with miR-424-5p mimics, miR-424-5p mimics negative control (miR-NC), miR-424-5p inhibitor, and miR-424-5p inhibitor negative control (miR-in-NC). **g** Wild type (WT) and Mutant type (MUT) SPTBN2 3' UTR sequences were cloned into pmirGLO luciferase reporter vector. **h** 293 T cells were co-transfected with miR-424-5p mimics, negative control, and luciferase reporters containing the wild type or mutated transcripts of SPTBN2 3′UTR as indicated. (*p < 0.05, **p < 0.01, ***p < 0.001, ****p < 0.0001. The data expressed as the mean ± SD).

Through the study on the dysregulation of miRNA, the rationale of aberrant SPTBN2 expression in endometrial cancer was investigated to be examined. Six miRNAs were predicted might be the potential upstream regulator of SPTBN2 by four bioinformatics websites (Targetscan7.2: https://www.targetscan.org/vert_72/; PITA: https://genie.weizmann.ac.il/pubs/mir07/index.html; miRmap: https://mirmap.ezlab.org/; miRanda: http://www.miranda.org/) (Fig. 6a). Combined with the six miRNAs expression in TCGA UCEC project via Genomic Data Commons Data Portal, only miR-195-5p, miR-497-5p, and miR-424-5p were downregulated in UCEC (Fig. 6b). To explore whether SPTBN2 was regulated by miR-195-5p, miR-497-5p, or miR-424-5p, the expression of SPTBN2 was detected after upregulating the candidate miRNAs; results indicated that only miR-424-5p resulted in a significant decrease of SPTBN2 mRNA expression (Fig. 6c). What’s more, the qRT-PCR analysis indicated that miR-424-5p expressed lower in 24 EEC tissues than in normal tissues (Fig. 6d), and was negatively correlated with SPTBN2 in EEC (*r* = −0.58, Fig. 6e). To further investigate, SPTBN2 was regulated by miR-424-5p, SPTBN2 expression was detected after down or up-regulating miR-424-5p, the results showed upregulated miR-424-5p could reduce the mRNA and protein levels of SPTBN2, while downregulated miR-424-5p increased SPTBN2 expression (Fig. 6f). Next, the direct binding site affinity between SPTBN2 3'-UTR and miR-424-5p was further confirmed by Dual luciferase reporting assay (Fig. 6g, h). These results suggested that miR-424-5p regulated SPTBN2 expression by directly binding to its 3'-UTR. Therefore miR-424-5p was finally selected as the candidate miRNA.

### MiR-424-5p is a tumor-suppressing miRNA downregulated in endometrial cancer

**Fig. 7:**
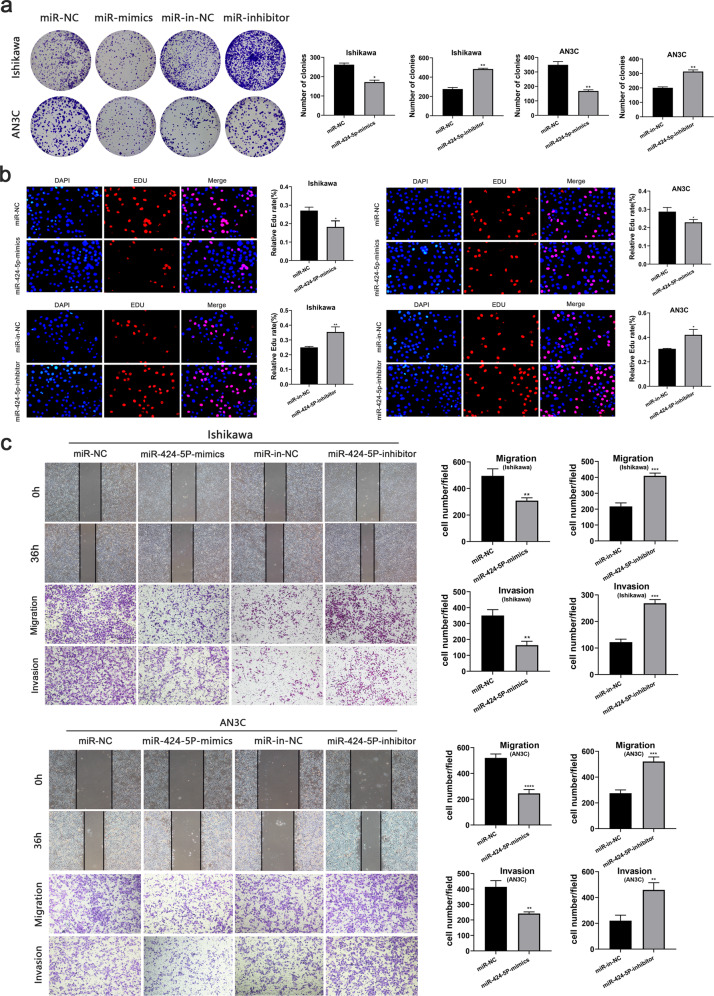
miR-424-5p is downregulated in UCEC and plays a tumor-suppressive role. **a**, **b** The effect of miR-424-5p on proliferation was detected by colony formation and Edu assays in UCEC cells. **c** Wound healing and Transwell assays were performed to show that miR-424-5p overexpression inhibited the migrated cells, whereas miR-424-5p depletion increased the cell movement in UCEC cells. (*p < 0.05, **p < 0.01, ***p < 0.001, ****p < 0.0001. The data expressed as the mean ± SD).

Since miR-424-5p was a regulator of SPTBN2, it’s biological function in UCEC was next explored. The colony numbers were significantly reduced by miR-424-5p mimics while increased by its inhibitor in UCEC cells (Fig. 7a). Moreover, the EDU experiment displayed the same effects as presented in CCK8 (Fig. 7b). There is no doubt that these data verified the essential role of miR-424-5p in regulating proliferation in endometrial cancer. By carrying the Wound healing and Transwell assay, we examined the influence of re-expression miR-424-5p on weakened metastasis in UCEC cells, but the ability was enhanced after transfected with miR-424-5p inhibitor (Fig. 7c). These results suggested that miR-424-5p functioned as a tumor suppressor in endometrial cancer.

### MiR-424-5p is involved in PI3K/AKT signaling pathway by targeting SPTBN2

**Fig. 8:**
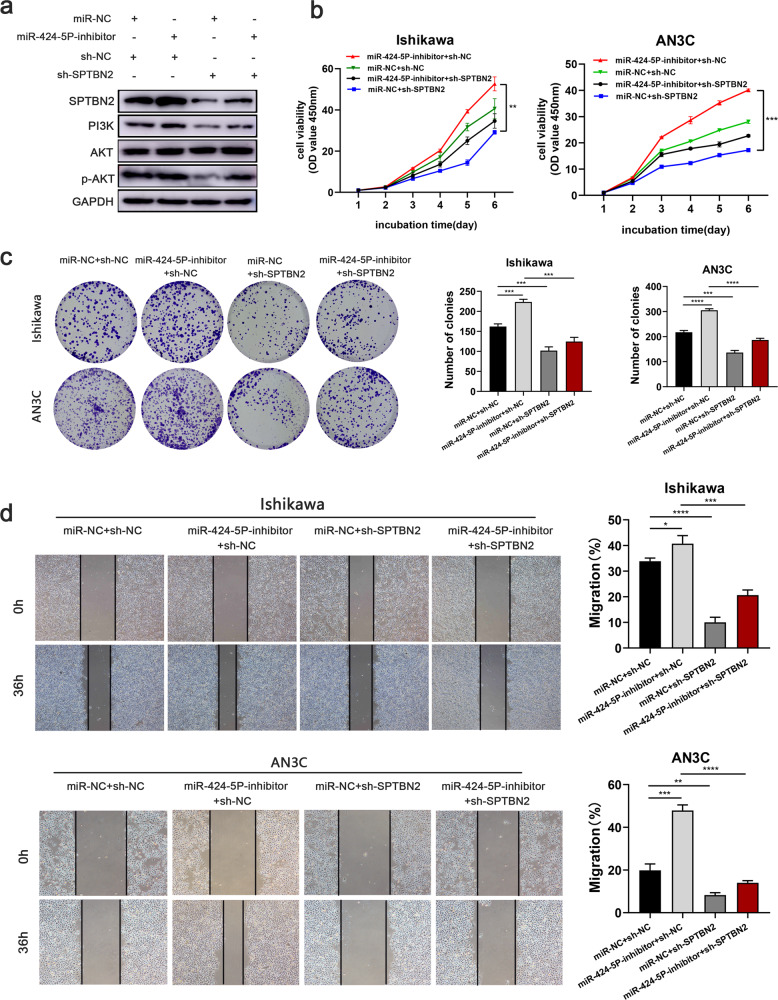
Downregulated expression of SPTBN2 could partly counteract the effect of miR-424-5p on UCEC cells. **a** Western blot analysis of SPTBN2 and PI3K/AKT signaling pathway-related markers of the rescue experiment in UCEC cells. **b**, **c** CCK8 and colony formation assays indicated that the effect of miR-424-5p on cell proliferation could be reversed by downregulated expression of SPTBN2. **d** Wound healing assay revealed that downregulated expression of SPTBN2 partially reverse the suppressive effect of miR-424-5p on metastasis (*p < 0.05, **p < 0.01, ***p < 0.001, ****p < 0.0001. The data expressed as the mean ± SD).

Rescue experiments were performed to investigate whether SPTBN2 was a function target of miR-424-5p, and if these effects could be regulated by miR-424-5p. After miR-NC + sh-NC, miR-424-5p inhibitor + sh-NC, miR-424-5p inhibitor + shSPTBN2, and miR-NC + shSPTBN2 were co-transfected into UCEC cells respectively. Western blot showed that the protein expression of SPTBN2 and the genes related to PI3K/AKT signaling pathway were enhanced after miR-424-5p was silenced, which could be partially attenuated by SPTBN2 knockdown (Fig. 8a). CCK8 and colony formation assays showed that silencing SPTBN2 could reverse the effect of miR-424-5p on proliferation (Fig. 8b, c). Meanwhile, wound healing assay showed that silencing SPTBN2 partially restored the effects of miR-424-5p on metastasis accordingly (Fig. 8d). These data suggested that silencing SPTBN2 expression could reverse the effect of miR-424-5p on cell growth and metastasis in endometrial cancer.

## Discussion

In our study, we primarily highlight the clinical significance and molecular mechanism of aberrant SPTBN2 expression. Currently, most studies have focused on the role of SPTBN2 in neurodegenerative diseases such as ataxia and cognitive impairment [10, 23]. Mutations in this gene always result in a form of spinocerebellar ataxia [24]. Moreover, SPTBN2 has been detected in more than 90% of malignant peripheral nerve schwannomas (MPNST), but not in normal peripheral nerve and benign neurofibromas. Subsequently, the possibility of SPTBN2 as a diagnostic biomarker for MPNST was verified [7]. What’s more, SPTBN2 was associated with poor prognosis in patients with colorectal cancer and was highly expressed in patients with distant metastasis, lymph node metastasis, and clinical advanced colorectal cancer, with statistically significant differences [8]. In recent studies, it has been confirmed that the mRNA and protein levels of SPTBN2 were upregulated in ovarian cancer, and which was involved in a variety of biological processes associated with tumorigenesis and was highly associated with poor prognosis of ovarian cancer [9]. In our study, we found SPTBN2 was significantly upregulated in human UCEC for the first time, and the high expression of SPTBN2 was related to poor prognosis, suggesting that SPTBN2 can be served as a powerful predictor of UCEC prognosis. In addition, molecular function tests suggested SPTBN2 was involved in the proliferation, migration, and invasion, which served as an oncogene in EEC both in vitro and in vivo. And it may be a potential therapeutic target for endometrial cancer patients.

Next, we explored the potential mechanism of highly expressed of SPTBN2 in the promotion of endometrial cancer occurrence and development. Our results indicated that SPTBN2 promoted UCEC metastasis by regulating many EMT-related genes. Epithelial–mesenchymal transition (EMT) is a biological process in which tumor-associated epithelial cells lose their epithelial characteristics and acquire a mesenchymal phenotype [25]. In the process of EMT, cell morphology, cell adhesion, and many cell pathways are changed, which increase the migration and invasion ability of tumor cells. Cancer cells invade the surrounding tissues and eventually lead to the progress of cancer metastasis [26–28]. An important molecular feature of EMT is the downregulation of E-cadherin. E-cadherin is considered a tumor suppressor, which can reduce the invasion and metastasis of tumor cells, and is often inhibited or degraded during transformation [29]. In our study, we observed the upregulation of the epithelial marker E-cadherin, while the mesenchymal markers N-cadherin, Snail, and Slug were downregulated after SPTBN2 knockdown in protein level. In conclusion, SPTBN2 may become a potential biomarker for the prognosis and treatment targets of UCEC.

Our investigation also demonstrated that CLDN4 served as an oncogene in UCEC. Interestingly, it was found that the expression of CLDN4 was positively correlated with SPTBN2. CLDN4 is a crucial member of the tight junction protein, although the role of CLDN4 in carcinogenesis remains controversial [16], many studies have revealed the carcinogenic functions of CLDN4, whose abnormal expression has been shown to contribute to tumor development [30], such as ovarian [15], breast [31], prostate [21], pancreatic [32] and gastric [33] cancers. CLDN4 is overexpressed in several tumors and has been suggested to have a carcinogenic role in cell proliferation, motility, invasion, and survival [34]. In addition, CLDN4 has been shown to be involved in epithelial–mesenchymal transformation (EMT) in gastric cancer and is the most important protein associated with lymphatic invasion and can be used as a prognostic marker [35]. A growing body of evidence shows that activation of the PI3K/AKT pathway could regulate the EMT process in a variety of cancer cells [29, 36, 37]. In our study, blocking SPTBN2 during the culture of UCEC cells reduced the expression of CLDN4 and PI3K/AKT-related genes. IP assay further indicated that SPTBN2 could directly interact with CLDN4.

CLDN4 knockdown could markedly inhibit cell proliferation, migration, and invasion through the inactivation of PI3K/AKT pathway via protein interaction. In addition, over-expressed CLDN4 could partially reverse the effects of SPTBN2 knockdown on tumor progression. All in all, our work proved that SPTBN2/CLDN4/PI3K/AKT axis might be a potential therapeutic target in UCEC.

Multiple studies have shown that miRNAs are involved in tumor progression and metastasis of a variety of human cancers [38, 39]. After performing the database and conducting a series of validation as mentioned, we identified miR-424-5p as an upstream regulator to inhibit SPTBN2 expression in endometrial cancer. Hsa-miR-424-5p is located on chromosome Xq.26.3. It is a member of the miR-16 family and can participate in cell cycle, proliferation, apoptosis, and other biological behaviors [40]. miR-424-5p is abnormally expressed in many types of tumors, such as thyroid cancer [41], intrahepatic cholangiocarcinoma [42], breast cancer [43], ovarian cancer [44], colorectal cancer [45], etc. However, it plays two different roles in different tumors: oncogene or tumor suppressor gene. At present, there is no relevant research in endometrial cancer. Our study suggested that overexpression of miR-424-5p significantly inhibited the cell growth and metastasis in UCEC, and miR-424-5p expression was negatively correlated with SPTBN2. In addition, rescue experiments confirmed that SPTBN2 could partially reverse the tumor-suppressive effect of miR-424-5p.

## Conclusion

In conclusion, our study revealed that the overexpression of SPTBN2 was closely associated with the poor prognosis in UCEC, and functioned as an oncogene promoted UCEC tumorigenesis through activation of the PI3K/AKT pathway by interacting with CLDN4. The newly confirmed key role of miR-424-5p/SPTBN2/CLDN4 axis may provide a basis for finding potential therapeutic targets of UCEC.

## Supplementary information

Supplementary Materials and methods

Supplementary Table.1

Supplementary Table.2

Supplementary Table.3

## Data Availability

For original data from xingshengyang@sdu.edu.cn..
