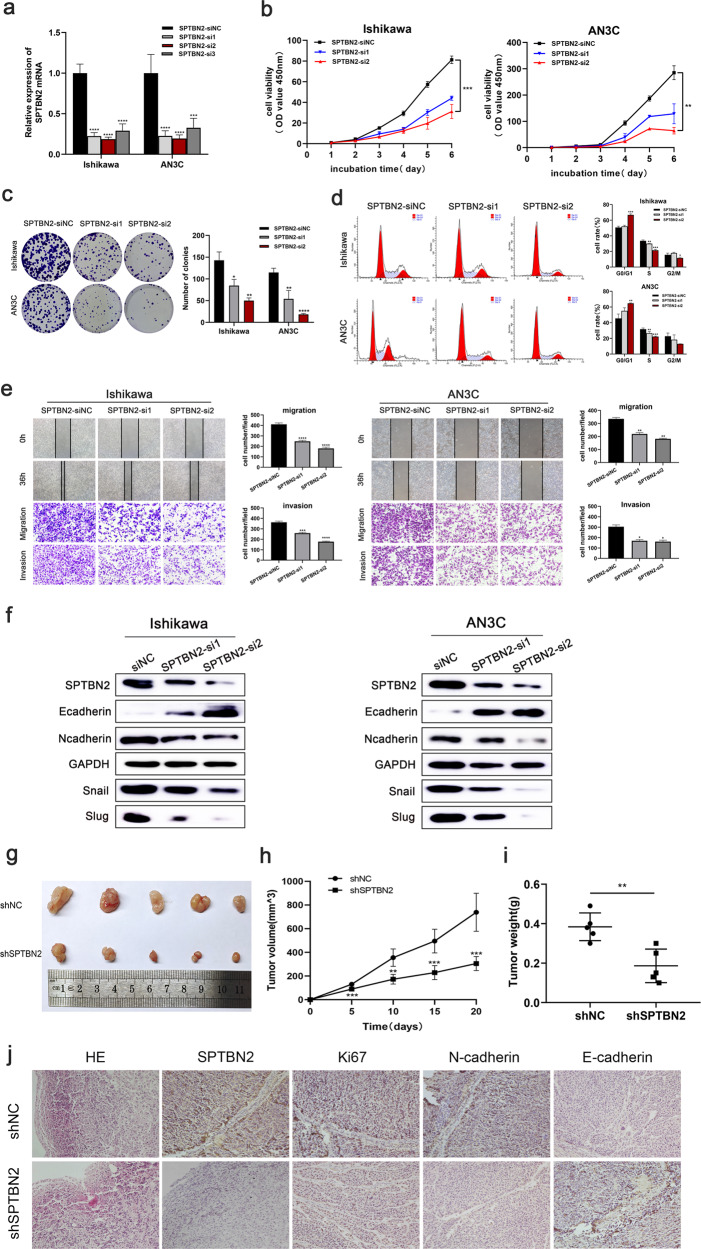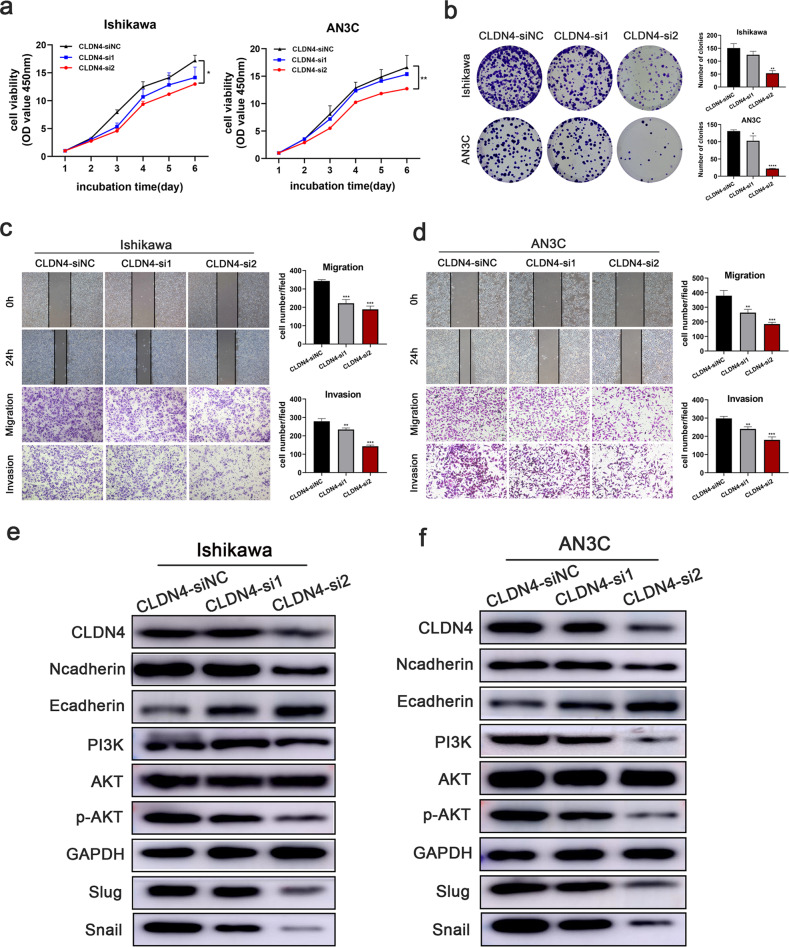# Correction: SPTBN2 regulated by miR-424-5p promotes endometrial cancer progression via CLDN4/PI3K/AKT axis

**DOI:** 10.1038/s41420-022-00934-5

**Published:** 2022-04-19

**Authors:** Pengling Wang, Ting Liu, Zhendan Zhao, Zhiling Wang, Shujie Liu, Xingsheng Yang

**Affiliations:** Department of Obstetrics and Gynecology, Qilu Hospital of Shandong University, Jinan, Shandong 250012 People’s Republic of China

**Keywords:** Endometrial cancer, Endometrial cancer

Correction to: *Cell Death Discov* 10.1038/s41420-021-00776-7, published online 9 December 2021

The original version of this article unfortunately contained errors in Figures 2 and 4. The authors apologize for the mistake. The corrected figures can be found below. The original article has been corrected.