# Supplementary material for: SPTBN2 regulated by miR-424-5p promotes endometrial cancer progression via CLDN4/PI3K/AKT axis
Source: Cell Death Discov. 2021 Dec 9;7:382. doi: 10.1038/s41420-021-00776-7 (PMC8660803; doi:10.1038/s41420-021-00776-7)
Supplement: Supplementary file 1 — Supplementary Materials and methods [file 41420_2021_776_MOESM1_ESM.docx]

**Materials and methods**

**Cell lines and cell culture**

The two human UCEC cell lines, Ishikawa and AN3C were obtained from the Key Laboratory of Gynecologic Oncology of Shandong Province which were tested and authenticated. The Ishikawa cell and the immortalized endometrial fibroblast cell were cultured in RPMI 1640 medium, while AN3C cell were cultured in DMEM/F12 medium. All the medium were supplemented with 10% fetal bovine serum(FBS;04-001-1A;Biological Industries,Israel), and 1% penicillin-streptomycin(P1400; Solarbio, China), and all cells were cultured in a sterile and humidified incubator at 37°C with 5% CO2.

**RNA extraction and qRT-PCR**

Total RNA were extracted from tissues and cells by TRIzol reagent (Invitrogen; CA, USA). total RNA was then reverse-transcribed into cDNA using the reverse transcription cDNA kit (Toyobo; Japan). RT-PCR analysis was performed on the Light Cycler Roche 480 PCR instrument. The mRNA relative expression was determined by the 2^-ΔΔCt^ method. Primer sequences are listed in Table3.

**Immunohistochemistry (IHC)**

Mouse tumor tissues were fixed in formalin, embedded in paraffin, and cut into 4μm thick sections. After incubated at 62°C for 2h, dewaxed and rehydrated with xylene and ethyl alcohol. Antigen retrieval was performed using citrate buffer(pH=6) at 97 °C for 15 min. 3% hydrogen peroxide was used to block endogenous peroxidase activity for 15 min at 37℃. Incubating sections with 5% normal goat serum in humidified box for 1 h at 37℃ could prevented nonspecific binding of antibodies. Then，sections were incubated with primary antibodies against SPTBN2, Ki67, E-cadherin, N-cadherin overnight at 4 °C. Next day, after incubated with biotin-labeled goat anti-rabbit IgG polymer for 30 min at 37℃, DAB solution was used to detect the signals before counterstaining with hematoxylin. Scoring of the immunohistochemistry experiments was performed using a semi-quantitative method with the investigator blinded as to the identity of the samples. The calculation formula was as follows: H-score = ΣPi (i + 1). Pi in the formula represents the percentage of positive cells in the total number of cells in one slide: 0-5% is 0 points, 6-25% is 1 point, 26-50% is 2 points, 51-75% is 3 points,> 75 % is 4 points. i represents the intensity of coloration, according to the depth of coloration under the microscope: 0 points for no coloration; 1 point for light yellow; 2 points for brown yellow; 3 points for tan; negative 0 for H-score (-), 1-4 are classified as weakly positive (+), 5-8 are classified as moderately positive (++), and 9-12 are classified as strongly positive (+++). The IHC results were evaluated independently by two experienced pathologists.

**Protein extraction and Western blotting**

Samples and cells were harvested and lysed in RIPA cell lysis buffer (Beyotime; Shanghai, China), place on ice to fully lyse for 30 minutes. Then centrifuged for 15 min at 12,000g,4°C. Collect the supernatant, the protein concentration was detected by BCA Protein Assay kit (Merck Millipore, USA). Proteins were separated by SDS-PAGE electrophoresis and transferred onto PVDF membranes (Merck Millipore, Burlington, MA, USA), Then the PVDF membranes were blocked by 5% skim milk for 1-2 h at room temperature and incubated with target primary antibodies SPTBN2(1:500; Santa Cruz biotechnology; sc-376487; mouse), CLDN4(1:500; Santa Cruz biotechnology; sc376643; mouse), Ecadherin(1:1000;Cell Signaling Technology;#14472;Rabbit), Ncadherin(1:1000; Cell Signaling Technology;#13116;Rabbit), Snail(1:1000; Cell Signaling Technology;#3879;Rabbit), Slug(1:1000; Cell Signaling Technology;#9585;Rabbit), PI3K(1:1000; Cell Signaling Technology;#4249;Rabbit), PAKT(1:1000; Cell Signaling Technology,#4060;Rabbit), AKT(1:1000; Cell Signaling Technology,#4691;Rabbit) and GAPDH(1:2000;Servicebio;GB11002;Rabbit) at 4°C overnight. Next day, the membrane was washed three times by TBST and incubated with corresponding secondary antibodies (1:2000; Abmart) at room temperature for 1h, Then protein bands were detected with ECL detection system (Amersham Imager 600; Boston). The relative protein level was calculated by Image J software.

**SiRNA and lentivirus transfection**

Small interfering RNA (siRNA) to knockdown SPTBN2, CLDN4, and corresponding controls were purchased from GenePharma (Shanghai, China). CLDN4 overexpression plasmids were purchased from WZ Biosciences (Shandong, China). INTERFERin (Polyplus, Shanghai, China) was used to transiently transfected cells to realized siRNA silencing, After 24–72h transfection, cells were harvested and lysed to evaluate the transfection efficiency. SPTBN2 knockdown lentiviruses were purchased from Genechem (Shanghai, China). When the UCEC stable cell line grew to 30 to 40% confluency they were infected with lentiviral expression vector (MOI: 20) and selected with 8ug/ul puromycin for about 1 week. The siRNA, plasmids and lentivirus sequences are detailed in Table 3.

**CCK8 and EDU assay**

For CCK8 and EDU analysis, cells were transfected with siRNA. Cell Counting Kit-8 (CCK8) (APExBIO, #K1018, Shanghai, China) was used to detect the cell proliferation ability. In a 96-well plate, 800-1000 cells per well were seeded into each well, and incubated in a 37℃ cell incubator for 24h. Proliferation rates were detected at 0, 24, 48, 72 and 96h after transfection, before detecting the optical density (OD), 10 μl CCK8 were added to each well and 1h incubation at 37 °C was continued, and OD reading was performed on a Varioskan Flash microplate reader (Thermo Scientific) at 450 nm following the manufacturer’s protocol. For EDU (RiboBio, #C10310, Guangzhou, China) analysis, the EDU solution was diluted with medium in 1:1000, and 4x10^3-1x10^5 cells per well were seeded in 96-well plate, cultured in EDU containing medium for 2h. Then cells were fixed by methanol at room temperature for 30 min after removing EDU containing medium, and 0.4% Tritonx cultured for 30min was used to increase cell membrane permeability. Images were acquired using a fluorescence microscope after washing and Apollo and Hoechst dye. The percentage of EDU positive cells is calculated as follows: (EDU stained cells/Hoechst stained cells) × 100%.

**Colony formation assay**

The transfected cells were seeded at a density of 500 cells/well in 12-well plates and then incubated for two weeks. Two weeks later, methanol was used to fix the cell, and 0.1% crystal violet solution was used to stain the cells. The number of formed colonies was photographed and counted under a microscope. Finally, we can calculate the colony formation rate.

**Flow cytometry assay**

The transfected cells were fixed with 90% Ethanol at -20℃ for 1h,washed twice with cold PBS, resuspended in 500ul PBS, and incubated with 20ul RNase A in water bath at 37℃ for 30min. filter with 400 mesh cell screen. Then added 400ul PI to resuspend the cells, and incubate for 30-60 minutes at 4℃ in the dark. Flow cytometry will be completed within 24 hours after staining. The cell cycle distribution of 10,000 cells was calculated using Beckman Coulter(Beckman, USA) Modfit LT software.

**Transwell assay**

Transwell assays were performed in Boyden chambers (8μm pores, BD Biosciences, USA) which were inserted into 24-well plates without or with Matrigel (BD Biosciences, USA). For the migration assay, The transfected cells (8×10^5-1×10^5 cells) in 200μl serum-free medium were placed in the upper chamber, and 700ul of culture medium supplemented with 20% FBS was placed in the lower chamber. After incubating at 37°C for 24h, cotton swabs were used to removed cells on the upper side of the filters, cells that had migrated to the lower surface of the filters were fixed with methanol for 20min and stained with 0.5% crystal violet for 20min. Then observed and counted under a light microscope. The conditions of the invasion assay were the same as the migration assay, except that the invasion chambers were coated with biocoat Matrigel.

**Wound healing assay**

The transfected cells were seeded into 6-well plates at a density of 1×10^5 cells/well , and then incubated at 37℃ overnight. When the cell fusion rate reached 90%, Scrap the cell layer at the bottom of the 6-well plate with the tip of the 200μL pipette, to creat a linear gap, and the exfoliated cells were washed off with PBS. Then, the cells were cultured with the fresh medium for 24 hours, and an inverted fluorescence microscope was used to get the cell migratory images. The migration rate= (healing width at 24h-healing width at 0 h)/healing width at 0 h.

**Animal experiment**

The animal experiments were approved by the the Laboratory Animal Ethical and Welfare Committee of Shandong University Cheeloo College of Medicine (Approval number: 19074). BALB/c mice (female, 4-6 weeks of age) were separated two groups randomly in animal experiment. Animals were separated randomly. The investigators were blinded to the group allocation during the experiment and when assessing the outcome. For subcutaneous xenograft tumorigenicity experiments (n = 5), Ishikawa cell line (15×10^6 cells/mice) transfected with shSPTBN2 or shNC were resuspended in 100 μl PBS and subcutaneously injected into the nude mice, The size of the tumors was measured by Vernier calliper weekly for 5 weeks. Tumor volumes were calculated by the following formula: V(tumor)=1/2 × d^2 × D (D= largest length, d= smallest width).The mice were sacrificed after 5 weeks, and tumors were removed for evaluation.

**Immunoprecipitation assay**

Proliferating cells with good growth state and 80% fusion degree in 10cm^2 dishes were harvested, collected in IP lysis buffer and place on ice to fully lyse for 30 minutes, then centrifuged at 12000 rpm for 15 min at 4 °C, and collected the supernatants, 100 µl supernatants were retained as input group. The remaining supernatants were incubated with SPTBN2 antibody overnight at 4°C. Next day, the precipitated protein was collected by protein A/G beads, washed and eluted with boiled IP buffer, which as IP group, and then Western blotting was performed.

**Luciferase reporter assay**

The 3′-UTR sequences of SPTBN2 containing wild-type or mutated miR-424-5p binding sites were synthesised and cloned into a pmirGLO luciferase reporter vector (Promega,USA). 293T cells were seeded in 24-well plates were co-transfected with miR-424-5p mimics or miR-NC with pmirGLO-WT-SPTBN2 or pmirGLO-MUT-SPTBN2 by using Lipofectamine 2000 (Invitrogen). 48 hours after transfection, cells were collected for frefly and Renilla luciferase activities detection using the Dual-Luciferase Reporter Gene Assay Kit (Beyotime,China) in accordance with the manufacturer’s protocol. The relative luciferase activity is the ratio of Firefly luminescence to the Renilla luminescence.
