# Supplementary material for: SPTBN2 regulated by miR-424-5p promotes endometrial cancer progression via CLDN4/PI3K/AKT axis
Source: Cell Death Discov. 2021 Dec 9;7:382. doi: 10.1038/s41420-021-00776-7 (PMC8660803; doi:10.1038/s41420-021-00776-7)
Supplement: Supplementary file 2 — Supplementary Table.1 [file 41420_2021_776_MOESM2_ESM.docx]

**Table.1**

| siRNAs/miRNA Oligo/shRNA/gene | | Sequence (5’-3’) |
| --- | --- | --- |
| SPTBN2-si1 | Sense | CCCAGGGAAAGAGUAUAGATT |
|  | Antisense | UCUAUACUCUUUCCCUGGGTT |
| SPTBN2-si2 | Sense | GCAGCCAGGAAUAUGUUCUTT |
|  | Antisense | AGAACAUAUUCCUGGCUGCTT |
| SPTBN2-si3 | Sense | CCCUGCAGAAAGAGAUUCATT |
|  | Antisense | UGAAUCUCUUUCUGCAGGGTT |
| CLDN4-si1 | Sense | CCACCCUCCUCUGGAUAUUTT |
|  | Antisense | AAUAUCCAGAGGAGGGUGGTT |
| CLDN4-si2 | Sense | GCUUUGUUCUUCCCUGGACTT |
|  | Antisense | GUCCAGGGAAGAACAAAGCTT |
| mir-424-5p mimics | Sense | CAGCAGCAAUUCAUGUUUUGAA |
|  | Antisense | CAAAACAUGAAUUGCUGCUGUU |
| mir-195-5p mimics | Sense | UAGCAGCACAGAAAUAUUGGC |
|  | Antisense | CAAUAUUUCUGUGCUGCUAUU |
| mir-497-5p mimics | Sense | CAGCAGCACACUGUGGUUUGU |
|  | Antisense | AAACCACAGUGUGCUGCUGUU |
| Negetive control | Sense | UUCUCCGAACGUGUCACGUTT |
|  | Antisense | ACGUGACACGUUCGGAGAATT |
| mir-424-5p inhibitor |  | UUCAAAACAUGAAUUGCUGCUG |
| Inhibitor NC |  | CAGUACUUUUGUGUAGUACAA |
| SPTBN2-LV |  | TGCATACTAAGTGGCAGAA |
| NC-LV |  | TTCTCCGAACGTGTCACGT |
| GAPDH | Forward primer | GCACCGTCAAGGCTGAGAAC |
|  | Reverse primer | TGGTGAAGACGCCAGTGGA |
| SPTBN2 | Forward primer | ACATCCACGCCGACAAGATT |
|  | Reverse primer | GCGCTGCGTCTTGATTCTTC |
| CLDN4 | Forward primer | CTGTGCCTTGCTCACCGAAAC |
|  | Reverse primer | CCCTCTAAACCCGTCCATCCA |
| mir-424-5p | Forward primer | GCCAGCAGCAATTCATGT |
|  | Reverse primer | TATGGTTTTGACGACTGTGTGAT |
| U6 | Forward primer | CAGCACATATACTAAAATTGGAACG |
|  | Reverse primer | ACGAATTTGCGTGTCATCC |
