# Supplementary material for: SPTBN2 regulated by miR-424-5p promotes endometrial cancer progression via CLDN4/PI3K/AKT axis
Source: Cell Death Discov. 2021 Dec 9;7:382. doi: 10.1038/s41420-021-00776-7 (PMC8660803; doi:10.1038/s41420-021-00776-7)
Supplement: Supplementary file 3 — Supplementary Table.2 [file 41420_2021_776_MOESM3_ESM.docx]

|  |  | **SPTBN2 expression** |  |  |
| --- | --- | --- | --- | --- |
| **Factors** | **Sample** | **Low** | **High** | **P value** |
| **Age** |  |  |  | 0.628298637 |
| ＜50 | 12 | 9(75%) | 3(25%) |  |
| ≥50 | 48 | 39(81.3%) | 9(18.7%) |  |
| **FIGO stage** |  |  |  | 0.196705602 |
| I+II | 48 | 40(83.3%) | 8(16.7%) |  |
| III-IV | 12 | 8(0.67%) | 4(33.3%） |  |
| **Differentiation grade** |  |  |  | 0.060079515 |
| High+Middle | 47 | 40(85.1%) | 7(14.9%) |  |
| Low | 13 | 8(61.5%) | 5(38.5%) |  |
| **Lymph node metastasis** |  |  |  | 0.8296381 |
| Positive | 6 | 5(83.3%) | 1(16.7%) |  |
| Negative | 54 | 43(79.6%) | 11(20.4%) |  |
| **Event** |  |  |  |  |
| Alive | 51 | 43(84.3%) | 8(15.7%) | 0.046753193***** |
| Dead | 9 | 5(55.6%) | 4(44.4%) |  |

**Table 2 Correlations between SPTBN2 expression and clinicopathologic characteristics of UCEC patients**
