# Supplementary material for: SPTBN2 regulated by miR-424-5p promotes endometrial cancer progression via CLDN4/PI3K/AKT axis
Source: Cell Death Discov. 2021 Dec 9;7:382. doi: 10.1038/s41420-021-00776-7 (PMC8660803; doi:10.1038/s41420-021-00776-7)
Supplement: Supplementary file 4 — Supplementary Table.3 [file 41420_2021_776_MOESM4_ESM.docx]

**Table 3 Correlations between CLDN4 expression and clinicopathologic characteristics of UCEC patients**

| Factors | Sample | CLDN4 expression  Low High | | P value |
| --- | --- | --- | --- | --- |
| Age |  | |  | 0.754000888 |
| ＜50 | 12 | | 9(75%) 3(25%) |  |
| ≥50 | 48 | | 38(79.2%) 10(20.8%) |  |
| FIGO stage |  | |  | 0.060079515 |
| I+II | 48 | | 40(83.3%) 8(16.7%) |  |
| III-IV | 12 | | 7(58.3%) 5(41.7%） |  |
| Differentiation grade |  | |  | 0.368064828 |
| High+Middle | 47 | | 38(80.9%) 9(19.1%) |  |
| Low | 13 | | 9(69.2%) 4(30.8%) |  |
| Lymph node metastasis |  | |  | 0.754000888 |
| Positive | 6 | | 5(83.3%) 1(16.7%) |  |
| Negative | 54 | | 42(77.8%) 12(22.2%) |  |
| Event |  | |  |  |
| Alive | 51 | | 43(84.3%) 8(15.7%) | 0.007435108* |
| Dead | 9 | | 4(44.4%) 5(55.6%) |  |
